# Acute urinary tract infection in patients with underlying benign prostatic hyperplasia and prostate cancer

**DOI:** 10.11604/pamj.2020.36.169.21038

**Published:** 2020-07-09

**Authors:** Musliu Adetola Tolani, Aisha Suleiman, Mudi Awaisu, Muhammad Mukhtar Abdulaziz, Ahmad Tijjani Lawal, Ahmad Bello

**Affiliations:** 1 Division of Urology, Department of Surgery, Ahmadu Bello University and Ahmadu Bello University Teaching Hospital, Zaria, Kaduna State, Nigeria,; 2 Department of Paediatrics, Ahmadu Bello University Teaching Hospital, Zaria, Kaduna State, Nigeria,; 3 Department of Medical Microbiology, Ahmadu Bello University and Ahmadu Bello University Teaching Hospital, Zaria, Kaduna State, Nigeria

**Keywords:** Benign prostatic hyperplasia, catheter-associated infections, prostate cancer, urinary tract infection

## Abstract

**Introduction:**

the occurrence of urinary tract infection in patients with obstructing prostate causes reduction in their health-related quality of life and overall well-being. The objective of this study was to determine the prevalence, risk factors and antimicrobial sensitivity pattern of pathogens causing urinary tract infection in patients with benign prostatic hyperplasia and prostate cancer.

**Methods:**

all patients who presented to our urology division with bladder outlet obstruction secondary to benign prostatic hyperplasia or prostate cancer between January 2016 and January 2019 were included. Information on age, co-morbid conditions, presence of an indwelling catheter, bacteriologic analysis, imaging findings and histological diagnosis were obtained and analyzed using SPSS version 20.

**Results:**

de-novo urinary tract infection occurred in 35.6% of patients while recurrent infection occurred in 5.9% of them. The most commonly isolated organisms were gram-negative bacteria with Escherichia coli, Klebsiella spp, Citrobacter spp and Aerobacter spp accounting for 62.2%, 27.0%, 8.1% and 2.7% respectively. Nitrofurantoin (64.3%), Ceftriaxone (46.3%) and Genticin (42.9%) were the three most sensitive antimicrobials to the organisms isolated. Only the presence of an indwelling catheter in the bladder was an independent predictor of urinary tract infection in the study population.

**Conclusion:**

about one-third of patients with benign prostatic hyperplasia and prostate cancer develop urinary tract infection. The predominant bacterial cause was Escherichia coli, which had a high degree of sensitivity to Nitrofurantoin. The presence of an indwelling catheter was the only independent predictor of this infection. Appropriate measures should be re-enforced to prevent the occurrence of catheter-associated infections.

## Introduction

Benign prostatic hyperplasia and prostate cancer are the most common predisposing pathologies for urinary tract infection in adults with bladder outlet obstruction [1]. The superimposition of this complication on patients affected by obstructing prostates worsens not just their already impaired health related quality of life and social well-being but also causes post-operative morbidity and imparts a huge strain in the financing of health services [2-4]. Organisms causing bacteriuria could be endogenous (peri-urethral colonization by host gastrointestinal tract flora) and exogenous (like cross-contamination in nosocomial settings). Commonly, peri urethral colonization and urethral ascent of the bacteria occurs to allow its access into the bladder [3]. Stasis of urine in bladder outlet obstruction further creates a medium for bacterial growth [2]. Type 1 pili mediated microbial adhesion to epithelium and cellular internalization then occurs leading to the formation of biofilm-like intracellular bacterial colonies [5].

The interplay of microbial virulence factors and host innate defense mechanisms trigger a neutrophil-mediated inflammatory response [4]. As a result, the epithelial cells containing some of the bacterial colonies are exfoliated to control the microbes. However, some of the bacteria in the other colonies that were unaffected by this cellular shedding are later released and then re-invade naïve already exposed bladder epithelial cells to cause infection. From this, quiescent intracellular reservoirs which occurs might re-emerge to cause recurrent infection [6]. The clinical presentation of urinary tract infection depends on the part of urinary tract involved, the offending microbe and the severity of the infection [7]. Elderly males, the peak age group with prostate enlargement, are more likely to experience severe infection [6]. Only few studies have focused on the risk factors for urinary tract infection in patients with benign prostatic hyperplasia and prostate cancer. Also, the treatment of urinary tract infection in these patients is usually empirical and sometimes ineffective in our setting [2, 6]. Thus, knowledge is required to direct effective prevention and treatment strategies for urinary tract infection in the patients. The aim of this study was to determine the prevalence, risk factors and antimicrobial sensitivity pattern of pathogens causing urinary tract infection in patients with benign prostatic hyperplasia and prostate cancer.

## Methods

**Study design:** a cross-sectional study was carried out in the urology division of our hospital between January 2016 and January 2019. Ethical approval was obtained from the Health Research and Ethics Committee of our institution. Male patients 40 years and above with bladder outlet obstruction secondary to benign prostatic hyperplasia or prostate cancer were included. Those who did not have microbiological analysis of their urine were excluded from the study.

**Data collection:** medical records of the patients were accessed for demographic data (including age of the patient), status of diabetes mellitus and the presence of an indwelling catheter. Data on trans-rectal ultrasonography-determined prostate volume and histological diagnosis of the patients were also extracted. Results of urine microscopy were obtained to identify the presence of red blood cells and pus cells as well as the gram-stain characteristic of the organism while data on culture and sensitivity of the same sample was gotten to identify the causative organism and their antimicrobial sensitivity pattern.

**Outcome measures:** significant microscopic pyuria and haematuria were defined as ≥ 3 white blood cells and ≥ 5 red blood cells per high power field respectively [8]. Urinary tract infection was defined as the presence of a positive urine culture [7]. The occurrence of more than one episode of the infection was described as recurrent urinary tract infection.

**Data analysis:** this was done using SPSS version 20. Mean and standard deviation, median and interquartile ranges, and frequency and percentages were used to present baseline data, prevalence rate of the infection, frequency of uropathogens and antibiotic sensitivity pattern as appropriate. Logistic regression was performed to identify independent risk factors for the infection. The p value ≤0.05 were regarded as statistically significant.

## Results

**Figure 1:**
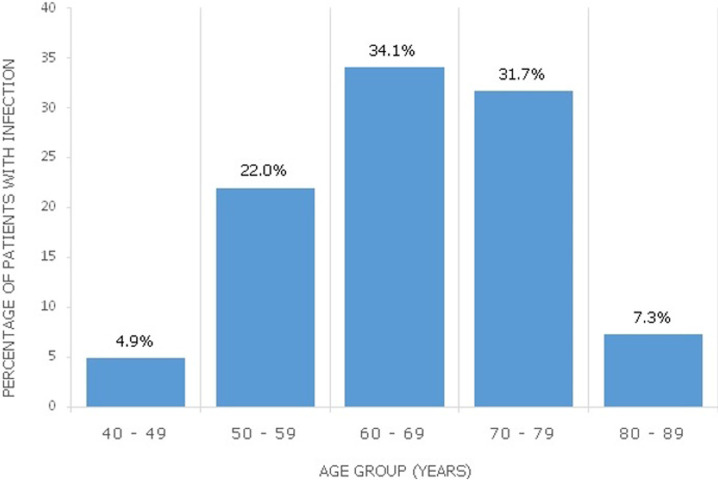
prevalence of urinary tract infection in different age groups of the study population

One hundred and sixty-six patients were diagnosed with benign prostatic hyperplasia or prostate cancer within the study duration. Fourty-eight of them were excluded due to incomplete data. A total of 118 patients were finally analyzed. Patient characteristics in the overall study population are displayed on Table 1. Just above one-fifth of them (22.0%) had prostate cancer. The mean age of patients with UTI was 64.6 ± 9.4 years and their age distributions are displayed on Figure 1. Microscopic findings on urine examination were significant pyuria in 71 (60.2%) and significant haematuria in 36 (30.5%). Significant pyuria had a sensitivity of 92.9% and specificity of 57.9% in predicting urinary tract infection. The rate of de-novo urinary tract infection was 42 (35.6%) with cause-specific prevalence of 33 (35.9%) and 9 (34.6%) in benign prostatic hyperplasia and prostate cancer respectively (p = 0.906). Recurrent urinary tract infection occurred in 7 (5.9%) of the entire study population.

**Table 1:** baseline characteristics of the study population (N = 118)

Variables	Values
Age, years	65.4 ± 9.5
Total Prostate Volume, grams	52.2 (20.5 - 280.7)
Diabetes Mellitus	17 (14.4%)
Indwelling Catheter	48 (40.7%)

Age is presented as Mean ± Standard Deviation, Total Prostate Volume as Median (Minimum - Maximum) and others as Number (%)

**Figure 2:**
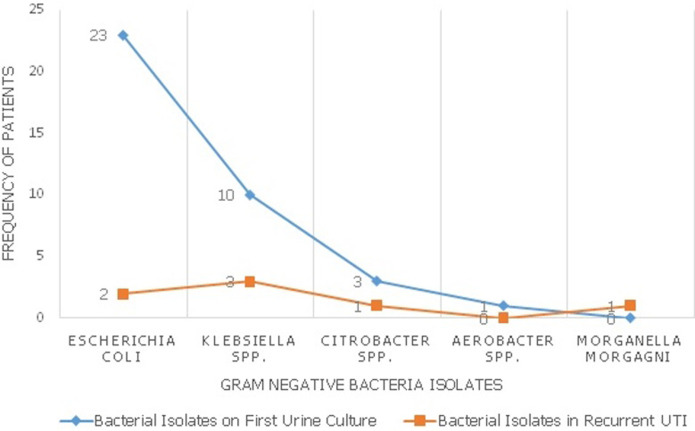
frequency of isolation of gram negative bacteria in the study population

**Figure 3:**
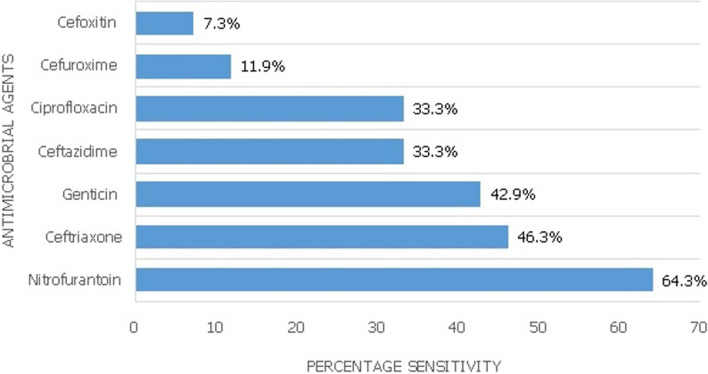
overall sensitivity of bacterial isolates to antimicrobial agents

Thirty-seven (88.1%) of the initial isolates were gram-negative organisms while 5 (11.9%) were gram-positive organisms. Figure 2 shows the distribution of the gram-negative microorganisms in the first urine culture of all patients and in those who developed recurrent urinary tract infection. Nitrofurantoin (64.3%), Ceftriaxone (46.3%) and Genticin (42.9%) were the three most sensitive antimicrobials to bacterial isolates cultured in this study (Figure 3). The sensitivities of specific bacterial isolates to the antimicrobials are shown on Table 2. Diabetic mellitus, hydronephrosis and indwelling urethral catheter were noted in 4 (9.5%), 8 (19.0%) and 28 (66.7%) of those with infection respectively. However, only the presence of an indwelling catheter in the urinary bladder was an independent predictor of urinary tract infection in the study population (p = 0.001) (Table 3).

**Table 2:** sensitivity of specific bacterial isolates on initial urine culture to the antimicrobial agents

Micro-organisms	AMC	CIP	CTR	CTZ	CFX	CXT	CFM	GTC	NFT
Escherichia coli	3 (13.0)	5 (21.7)	9 (39.1)	3 (13.0)	0 (0.0)	2 (8.7)	0 (0.0)	7 (30.4)	16 (69.6)
Klebsiella spp.	4 (40.0)	5 (50.0)	3 (30.0)	6 (60.0)	2 (20.0)	0 (0.0)	0 (0.0)	6 (60.0)	4 (40.0)
Citrobacter spp.	1 (33.3)	2 (66.7)	1 (33.3)	2 (66.7)	0 (0.0)	0 (0.0)	0 (0.0)	1 (33.3)	1 (33.3)
Aerobacter spp.	0 (0.0)	0 (0.0)	0 (0.0)	1 (100.0)	0 (0.0)	0 (0.0)	0 (0.0)	0 (0.0)	0 (0.0)
*S. aureus	2 (40.0)	1 (20.0)	4 (80.0)	1 (20.0)	2 (40.0)	1 (20.0)	0 (0.0)	2 (40.0)	4 (80.0)

AMC: Amoxicillin-Clavulanate, CIP: Ciprofloxacin, CTR: Ceftriaxone, CTZ: Ceftazidime, CFX: Cefuroxime, CXT: Cefoxitin, CFM: Cefixime, GTC: Genticin, NFT: Nitrofurantoin

*S. aureus: Staphylococcus aureus

All values are expressed as Number (%)

**Table 3:** possible predictors of urinary tract infection in the study population

Predictors	p-value
Age	0.809
Prostate Volume	0.860
Diabetes Mellitus	0.473
Indwelling Catheter	0.001
Prostate Cancer Presence	0.644
Hydronephrosis	0.449

## Discussion

Lower Urinary Tract Obstruction can result from pathologies in the region of the prostate, urethra or bladder neck. According to Asafo-Adjei *et al*. nearly 9 in 10 cases (86.7%) results from prostate abnormalities with around four-fifth of these due to benign prostatic hyperplasia [2]. Our study further confirms the predominant burden of the latter in the aetiology of bladder outlet obstruction (prostate cancer: 22.0%, benign prostatic hyperplasia: 78.0%). The rate of urinary tract infection in this study (35.6%) is within the range of 28-54% documented by Heyns in his systematic review of bacteriuria in patients with underlying urological abnormalities [1]. A higher rate of 76.6% was however reported in another study [2]. The higher rate of catheterization in their patients (83.5%) compared to our patients (66.7%) could have accounted for their increased infection burden. However, our study showed no difference in the rate of infection between patients with benign prostatic hyperplasia and those with prostate cancer (35.9% versus 34.6% respectively, p=0.906). The prevalence of UTI in our patients increased from 4.9% at 40-49 years to a peak occurrence of 34.1% at 60-69 years. This might not be unconnected with age-related decrease in prostatic fluid acidity and zinc associated antimicrobial factor [3].

The findings of this study which revealed that gram-negative organisms were isolated from the urine culture of most patients during the first episode of acute urinary tract infection (88.1%) is in consonance with the work of Prakasam *et al*. who documented that 96.7% of the bacteria isolated from the urine of their patients were gram negative [9]. This observation of predominant gram-negative bacteria cause of urinary tract infection in benign prostate hyperplasia and prostate cancer patients could guide the choice of empirical antimicrobial therapy in them. Specifically, *Escherichia coli, Klebsiella spp* and *Citrobacter spp* represented 62.2%, 27.0% and 2.7% of the gram-negative organisms isolated during the infection. This is close to the figures of 59.6%, 25.8%, 1.1% respectively, for the same organisms, observed by Gyasi-Sarpong *et al*. in another study [10]. This could mean that host intestinal bacterial flora was the likely source of the microbes and its pili and fimbriae might be a major role player for their virulence in the urinary tract [11]. The prevalence of *Escherichia coli* infection in the urinary tract might be particularly high as urinary tract abnormalities, either structural or functional, might promote infection with its strains that usually have lower p-fimbrial adhesive properties under normal conditions [1]. On the other hand, the isolation of *Staphylococcus aureus* (11.9%) as the only gram positive organism in the entire study population could be attributed to the use of urethral instrumentation in these patients [7].

Recurrent urinary tract infection occurred in 5.9% of the entire study population. Persistence of the micro-organisms following treatment or the occurrence of a new infection could be responsible for this [4]. Notwithstanding, the initiation of effective therapy following the first presentation of urinary tract infection could have accounted for the marked reduction in the proportion of those who developed a second infection. Pourmand *et al*. also observed a reduction in the rate of infection following surgical treatment of benign prostatic enlargement, thus, supporting our findings [12]. Overall, the bacterial isolates had a relatively higher sensitivity to Nitrofurantoin (64.3%) compared to other antimicrobials. Biswas and colleagues also observed that the organisms cultured from the urine were sensitive to Nitrofurantion in almost three-quarters (73.6%) of the cases [11]. This could be due to the narrowness of its spectrum of antimicrobial activity, its distribution in tissues and its indications for use [11]. It was also observed that *Staphylococcus aureus, Escherichia coli, Klebsiella spp*. and *Citrobacter spp*. showed the highest sensitivity to Nitrofurantoin/Ceftriaxone (80% each), Nitrofurantoin (69.6%), Ceftriaxone/Genticin (60.0% each) and Ceftriaxone/Ciprofloxacin (66.7% each) respectively. The high degree of susceptibility of *Staphyloccus aureus* to Nitrofurantoin and *Klebsiella spp* to Genticin has been documented in the previous study [11]. A striking finding in this study is the relatively low susceptibility of *Escherichia coli*, the most commonly isolated bacteria in urinary tract infection to Ciprofloxacin which is the most commonly prescribed empirical antimicrobial therapy for urinary tract infection. This calls for the revision of this antimicrobial to a more effective drug for treatment.

The predisposing factors for urinary tract infection depends on the patient population [13]. In this study, the confirmation of diabetes mellitus in nearly one-tenth (9.5%) of the patients with infection suggests that the high glucose level in their urine could serve as a medium for bacterial growth [7]. Similarly, the presence of hydronephrosis in about 1 in 5 patients (19.0%) with infection also suggests the need to exclude abnormalities of the upper urinary tract during their evaluation in order to prevent recurrence of the infection [4]. However, the only independent predictor of urinary tract infection in our study was the presence of an indwelling catheter in the urinary bladder (p = 0.001). This finding further confirms the result of Asafo-Adjei *et al*. who documented the same risk factor for urinary tract infection in patients with bladder outlet obstruction [2]. Released host fibrinogen deposited on indwelling catheters facilitates bacterial adhesion to its surface to establish a biofilm, which is more resistant to either immune-mediated or antimicrobial-driven bacterial clearance [6]. The duration of catheterization further worsens infection risk as Storme *et al*. noted that for every additional day of catheterization in men, infection risk increases by 3-4% [13]. Therefore, frequent catheter toileting and catheter change should be done in order to reduce this risk in patients with benign prostate hyperplasia and prostate cancer in our setting.

The limitations of this study include its retrospective nature, the lack of urine culture for anaerobes and non-inclusion of other new generation, albeit costly antimicrobials, during the urine culture.

## Conclusion

The prevalence of urinary tract infection in patients with benign prostatic hyperplasia and prostate cancer were 35.9% and 34.6% respectively. *Escherichia coli* was the most commonly isolated microbiological agent, and Nitrofurantoin, with a high sensitivity against the organisms, should be considered in the empirical treatment of the infection. The presence of an indwelling urethral catheter was the only independent predictor of this infection. Thus, preventive measures should be instituted to prevent catheter-associated infections in these patients.

### What is known about this topic

- Organisms causing urinary tract infection could be endogenous or exogenous;
- Superimposition of urinary tract infection on patients with obstructing prostates worsens their health related quality of life;
- Empirical treatment of urinary tract infection in these patients is sometimes ineffective in our setting.

### What this study adds

- In patients with obstructing prostates, de-novo and recurrent urinary tract infection occurred in 35.6% and 5.9% respectively;
- Nitrofurantoin should be considered in the empirical treatment of the infection in low resource settings;
- Active measures should be taken to prevent catheter-associated infections in these patients.
